# Differential Micro RNA Expression in PBMC from Multiple Sclerosis Patients

**DOI:** 10.1371/journal.pone.0006309

**Published:** 2009-07-20

**Authors:** David Otaegui, Sergio E. Baranzini, Ruben Armañanzas, Borja Calvo, Maider Muñoz-Culla, Puya Khankhanian, Iñaki Inza, Jose A. Lozano, Tamara Castillo-Triviño, Ana Asensio, Javier Olaskoaga, Adolfo López de Munain

**Affiliations:** 1 Multiple Sclerosis Unit, Biodonostia Institute, San Sebastián, Spain; 2 Neurology Department, University of California San Francisco, San Francisco, California, United States of America; 3 Intelligent Systems Group, Computer Science Faculty, University of the Basque Country, San Sebastián, Spain; 4 Neurology Department, Hospital Donostia, San Sebastián, Spain; Max Planck Institute for Evolutionary Anthropology, Germany

## Abstract

Differences in gene expression patterns have been documented not only in Multiple Sclerosis patients versus healthy controls but also in the relapse of the disease. Recently a new gene expression modulator has been identified: the microRNA or miRNA. The aim of this work is to analyze the possible role of miRNAs in multiple sclerosis, focusing on the relapse stage. We have analyzed the expression patterns of 364 miRNAs in PBMC obtained from multiple sclerosis patients in relapse status, in remission status and healthy controls. The expression patterns of the miRNAs with significantly different expression were validated in an independent set of samples. In order to determine the effect of the miRNAs, the expression of some predicted target genes of these were studied by qPCR. Gene interaction networks were constructed in order to obtain a co-expression and multivariate view of the experimental data. The data analysis and later validation reveal that two miRNAs (hsa-miR-18b and hsa-miR-599) may be relevant at the time of relapse and that another miRNA (hsa-miR-96) may be involved in remission. The genes targeted by hsa-miR-96 are involved in immunological pathways as Interleukin signaling and in other pathways as wnt signaling. This work highlights the importance of miRNA expression in the molecular mechanisms implicated in the disease. Moreover, the proposed involvement of these small molecules in multiple sclerosis opens up a new therapeutic approach to explore and highlight some candidate biomarker targets in MS.

## Introduction

Multiple sclerosis (MS) is a demyelinating disease of the central nervous system (CNS). It begins most commonly during late adolescence, young adulthood, or mid-life, and it is one of the most incapacitating diseases in this age range.

MS causes attacks of neurological dysfunction (loss of vision, difficulty in walking or moving a limb, vertigo, loss of sensation) or progressive dysfunction in these same areas. These “attacks”, also known as relapses, typically last for a few days, and resolve spontaneously. However, patients may not always completely recover from an attack and are sometimes left with a disability. Although most patients experience attacks with little or no progressive disability, called recurrent remittent (RR) forms, approximately 10–15% have progressive symptoms from onset, called primary progressive forms. Furthermore, more than 80% of patients that debut with RR will ultimately develop progressive symptoms after a prolonged period of exacerbations, usually after 10–20 years.

Etiologically, MS is a complex disease in which both genetic and environmental factors play a role. The genetics of MS are also complex without a clear inheritance pattern. The most relevant candidate genomic region is the HLA system [1]–[3], although several other genes are currently being described as important risk factors involved in MS, as for example IL2RA [4] or IL7R genes [5].

Gene expression profiling has been a useful tool to provide information about the molecular pathways involved in MS pathogenesis [6]–[8]. Several new studies have identified different expression patterns between relapses and remission [9], [10] suggesting that this clinical distinction of two states of the disease also has a molecular correlation.

Small non-coding RNA molecules (microRNA or miRNA) are a gene expression and protein synthesis modulating mechanism that has been recently identified in several species ranking from worms to humans. These miRNA are single-stranded RNA molecules of about 20–25 nucleotides (nt) encoded by nuclear genes (70–150 nt) and highly conserved among species. These genes are not translated into proteins but are processed from primary transcripts (called pri-miRNA) to short stem-loop structures called pre-miRNA and finally to functional miRNA. The expression pattern of miRNA varies over time and between tissues. These mature miRNA molecules are partially complementary to one or more mRNA sequences and they function through sequence-specific down-regulation of their target mRNA via mRNA degradation or inhibition of translation [11]. Initial computational analysis suggested there were more than 500 validated human miRNA [12], [13], although in the public database (mirbase) around 700 were proposed in October 2008.

It has been predicted that miRNAs may regulate around 30% of all cellular mRNA suggesting that these molecules play a critical role in virtually all cellular functions [14].

Although dysregulation of miRNA expression has been characterized mostly in cancer, it has recently been studied in many other diseases. Specifically, miRNAs have been proposed as a regulator of immune cell development [15], playing a role in the inflammatory response [16], and as a key player in the pathogenesis of neurodegenerative diseases [17].

We are reporting our study of the expression of 364 miRNA in samples from MS patients during a relapse and during remission, along with healthy controls, with the aim of understanding the regulatory mechanisms of these stages.

## Materials and Methods

### Recruitment of individuals

All patients were recruited in the Neurology Department of Hospital Donostia, located in the region of Gipuzkoa (Basque Country, Spain). The study was approved by the local institutional review board and all the samples were obtained with the written informed consent of the subjects. The patients were diagnosed as having MS according to the Mc Donald Criteria [18], [19].

In a first group (Group A), 21 blood samples were obtained: 9 from patients in remission, 4 from patients during a relapse before the administration of steroids and 8 from healthy volunteers. Total RNA, including miRNA, was extracted from these samples to carry out the study. Demographic data of the individuals studied can be found in Table S1.

**Figure 1:**
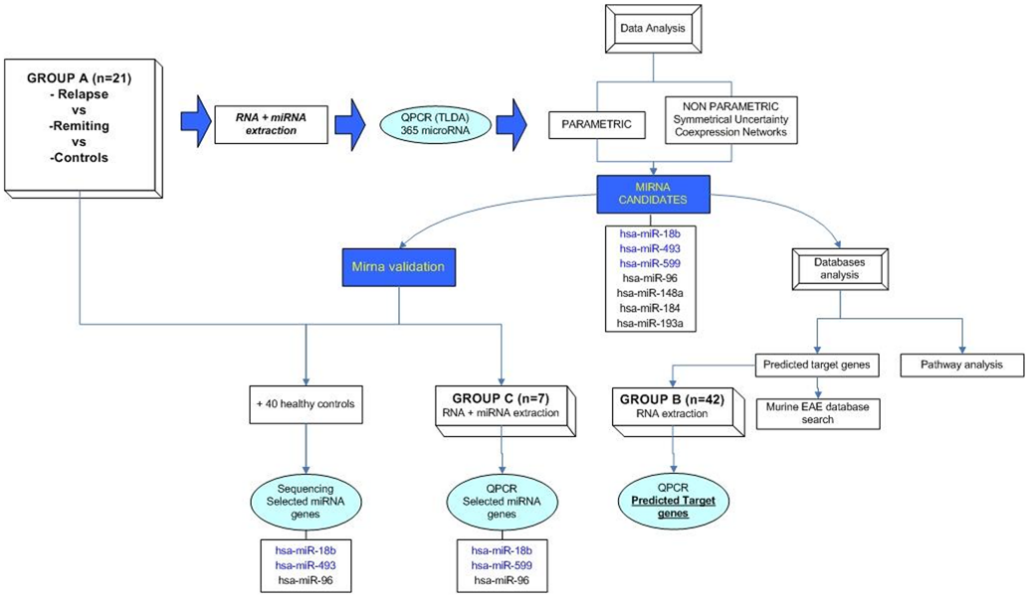
Workflow of the different approaches used in the work. The samples groups are specified and the selected genes are listed.

Samples were collected from two other non-related groups to validate independently some of the results obtained. Figure 1 summarizes the groups and the methods used:

- Group B: mRNA was obtained from 27 patients (14 during remission and 13 during relapse) and from 15 controls.
- Group C: miRNA was extracted from 7 patients (4 during relapse and 3 during remission) and from 7 healthy controls.

Blood extraction was always performed in the early morning and RNA extraction was carried out no more than 2 hours after the blood was collected and during this time was kept at 4°C. In all the cases, 10 ml of blood were collected in EDTA tubes by venipuncture.

### RNA extraction, reverse transcription (RT) and quantitative PCR (qPCR)

In groups A and C, total RNA was extracted from blood using the *Ambion Leucolock* kit (AM1923) working with the alternative protocol so as to keep the small RNA fraction.

The RNA obtained was quantified in triplicate using a NanoDrop spectrophotometer (NanoDrop Technologies, USA). A common bias in the interpretation of the miRNA profiles from whole blood may be introduced by the high concentration of miRNA from erythrocytes [20]. In our study we avoided such a bias by isolating PBMC in a filter prior to RNA purification (see *Ambion Leucolock* kit protocol).

cDNA was synthesized from total RNA using a Multiplex RT for Taqman array kit (Applied Biosystems, Foster City, CA). Briefly, this kit consists of 8 pre-defined RT primer pools containing up to 48 RT primers each. Each of these 8 pools contains the same endogenous controls (RNU48). This technology has been developed to detect only full length mature miRNA but not their precursors or their partially-degraded products.

We performed qPCR using the Taqman® Low Density Array (TLDA) Human MicroRNA Panel v1.0 from Applied Biosystems (see table S2 for a map of this array). This TLDA included 365 miRNA assays plus two selected endogenous controls. The qPCR was performed using an Applied Biosystems 7900 Sequence Detection System. Ct values were determined using the automatic threshold in *RQ manager v1.1* analysis software.

Two normalization steps were used: the first normalization consisted in loading the same quantity of template RNA in each well and the second in normalizing the data against an endogenous gene. This endogenous control (RNU48) was chosen for this study as the least variable of all endogenous genes included in the TLDA assays. Consequently, data was normalized to RNU48, using the values of each of the 8 pools, i.e. each gene pool was normalized against the endogenous gene that was converted to cDNA in the same pool, to avoid introducing bias in the results.

Relative quantification of miRNA expression was calculated with the 2^-ddCT^ method (Applied Biosystems User Bulletin N° 2 (P/N 4303859)). Quality of the data and quantification was computed using Real-Time Statminer© software (www.integromics.com). This software performs a moderate t-test between the groups (relapse, remitting and control) and corrects them using the Benjamini-Hochberg algorithm [21] with the False Discovery Rate (FDR) set at a value of 5%.

Samples from group B belong to an ongoing cohort collected by our group. These samples were extracted using the Versagene ^TM^ Kit (Gentra, Minneapolis, USA). This method entails the loss of the small molecules of RNA, i.e. miRNA.

### Statistical data analysis

A non-parametric analysis that complements the classical t-test analysis was performed trying to reveal alternative results over the low number of available samples. We compared the expression patterns of our three groups by pairs: relapse vs remitting, relapse vs control and remitting vs control. To accomplish this task, a non-parametric ranking method called Symmetrical Uncertainty (*SU*) sorts all the miRNA according to their statistical relevance over each of the three comparisons using the following coefficient,
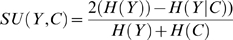
where *Y* is the predictive variable (in our case, each miRNA), *C* is the class label to be predicted (depending on the comparison carried out, it takes two of the following three values: remitting, relapse and control), *H*(*Y*) is the entropy of *Y* and *H*(*Y*|*C*) is the conditional entropy of *Y* given *C* [22].

The *SU* ranking is based on the mutual information between each miRNA expression level and the phenotype label. Being a univariate coefficient, it measures the uncertainty reduction of the class variable *C* when the expression value of a miRNA (denoted as *Y* in the above formulation) is known. As the *SU* metric only takes discrete/categorical variables, the DCT expression of each miRNA was first discretized into three intervals by using an equal width discretization method.

In order to get a multivariate view of the experimental data, we built co-expression networks to investigate the possible regulations within two out of our three comparisons (relapse *vs* remitting and remitting *vs* control). For this purpose we borrowed a technique for building gene interaction networks [23] and applied it to our DCT expression data. We used an algorithm that makes use of three main components to find reliable dependences from data: a bootstrap re-sampling algorithm, a supervised Bayesian network classifier and a dimensionality reduction technique. The algorithm's construction scheme is focused on finding highly reliable dependences from raw data. The bootstrap step re-samples the original data *B* times, obtaining *B* similar datasets. For each sampled dataset a dimensionality reduction step is made using the correlation-based filter selection approach (CFS) [24]. The CFS returns sets of relevant features that show a high degree of correlation with the class label while the redundancy degree among them is kept as low as possible. Each sampled dataset is projected to contain only the selected features and afterwards a *k*-dependence Bayesian [25] network classifier is induced from that data. All the identified probabilistic dependencies between pairs of nodes in the *B* final classifiers are stored. Note that the dependencies with respect to the supervised variable are not taken into account.

The algorithm's output is a hierarchy of probabilistic dependencies found during the whole process. When a cut-off threshold *T* is set, it is possible to retrieve a graphical structure in which only those probabilistic conditional dependencies that have been configured at least *T* times are displayed. Each arc in the final structure is associated with a robustness value which reflects the number of times the arc is configured in the different bootstrap re-samplings.

We perform a total of 10,000 re-samplings with their corresponding CFS and *k-*DB data mining techniques. The value of *k* in *k*-DB was set to four, keeping to the value suggested in the original work.

### Sequence of the miRNA genes

The miRNA genes were amplified by PCR (primers sequences available on request) and the PCR product was sequenced in an ABI3130 automatic sequencer (Applied Biosystems) using Bigdye v3.1. The used primers were designed based on the mirbase [13], [26], [27] sequence information and using the Generunner software (www.generunner.com). Group A samples (n = 21) were analyzed as well as 40 healthy controls. These healthy controls came from a cohort recruited in our group to test sequencing results, all the samples comes from healthy volunteer donors without neurological symptoms.

### Validation of the target genes

We studied an independent set of 42 samples (group B). The expression of predicted targets of the identified miRNAs was analyzed by qPCR using SYBRgreen as fluorescent and pre-designed primers from geneglobe (www.geneglobe.com). The assay codes can be found in Table 1. The data were analyzed using the same software and the same methodology described above, using as the endogenous gene GAPDH.

**Table 1:** Top 10 genes from the SU analysis for each of the three comparisons.

	Relevance as Symmetrical Uncertainty					
	Relap vs. Rem		Rem vs. CON		Relap vs. CON	
	gene	SU	gene	SU	gene	SU
**1**	hsa-miR-542-5p	0.5277	has-miR-96	0.4832	***hsa-miR-599***	0.8651
**2**	hsa-miR-376a	0.4921	hsa-miR-30a-5p	0.2989	***hsa-miR-18b***	0.6416
**3**	***hsa-miR-18b***	0.4048	hsa-miR-30e-5p	0.2959	hsa-miR-423	0.6367
**4**	hsa-miR-34c	0.4039	***hsa-miR-599***	0.2959	hsa-miR-125b	0.5738
**5**	hsa-miR-489	0.4039	hsa-miR-193a	0.2959	hsa-miR-383	0.5392
**6**	hsa-miR-554	0.4039	hsa-miR-337	0.2959	hsa-miR-509	0.5392
**7**	hsa-miR-600	0.4039	hsa-miR-449b	0.2591	hsa-miR-30e-5p	0.5392
**8**	hsa-miR-652	0.4039	hsa-miR-184	0.2477	hsa-miR-487b	0.5167
**9**	hsa-miR-214	0.3863	hsa-miR-328	0.2283	hsa-miR-222	0.4970
**10**	hsa-miR-328	0.3729	hsa-miR-146b	0.2238	hsa-miR-127	0.4965

Genes in red were also significant in the corrected t-test.

### Individual validation of the miRNA expression

Validation of the expression of the selected miRNA genes was performed in an independent set of 14 samples (Group C). The qPCR was performed in a 7900 sequence detection system using pre-designed Taqman probes (Applied Biosystems).

## Results

We used qPCR to study the expression of 364 miRNAs in samples from 4 MS patients during a relapse and from 9 patients during remission. We also analyzed 8 healthy controls.

On average, 45% of the miRNAs analyzed were expressed in any given sample. Differences in the expression of the miRNAs was tested between the different groups; relapse vs remitting, remitting vs controls, MS vs controls and relapse versus controls.

Although several miRNAs reached nominal significance in the t-test, only three remained significant after correction for multiple testing (with an FDR threshold of 5%) in all three pairwise comparison sets.

**Figure 2:**
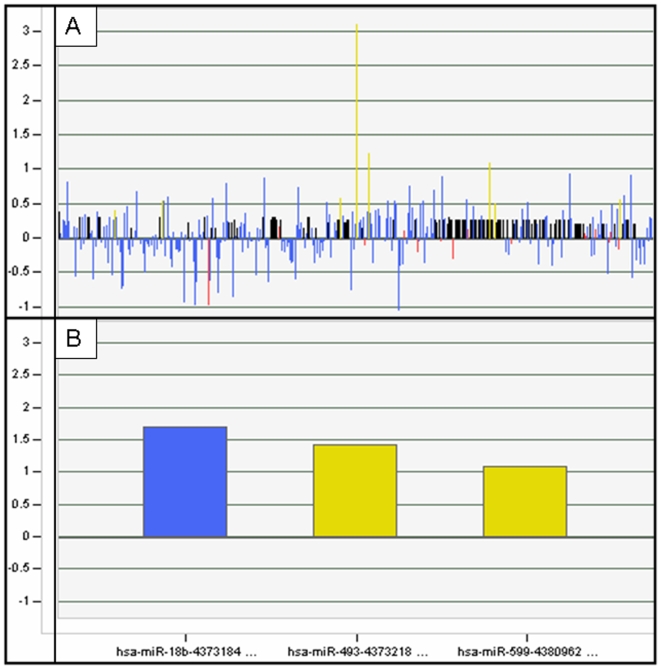
The charts show the log10 expression relative quantification values of 365 miRNA genes between Relapse (target) and control (calibrator) groups. A: this chart shows the values from the 361 genes that not passed the False discovery rate threshold (p = 0.05). B: Shows the values from the three genes that pass the false discovery rate threshold. Yellow: Calibrator not detected. Black: No detection. Red: Target not detected. Blue both (target and calibrator) detected.

The transcript ***hsa-miR-18b*** showed increasing expression in the relapsing group when compared to controls (RQ: 52.1). The transcripts ***hsa-miR-493*** and ***hsa-miR-599*** showed reliable expression in the relapsing group whereas they were not detected in controls. These two miRNA were also expressed in the remission group but did not reach any statistical significance in the comparisons. (See fig. 2, DDCT and p values of all the comparisons are provided in table S3)

In order to complement the information of the classical statistical analysis, we calculated the symmetrical uncertainty (SU) correlation degree of each miRNA with respect to the class phenotype, providing a ranked list of all miRNAs. The top ten miRNA emerging from these rankings are shown in Table 1.

Rankings were made for three different comparisons; relapse versus remitting, remitting versus controls and relapse versus controls. Highlighted in red are the significant miRNA found in the previous analysis (Complete data analysis could be found as table S4).

**Figure 3:**
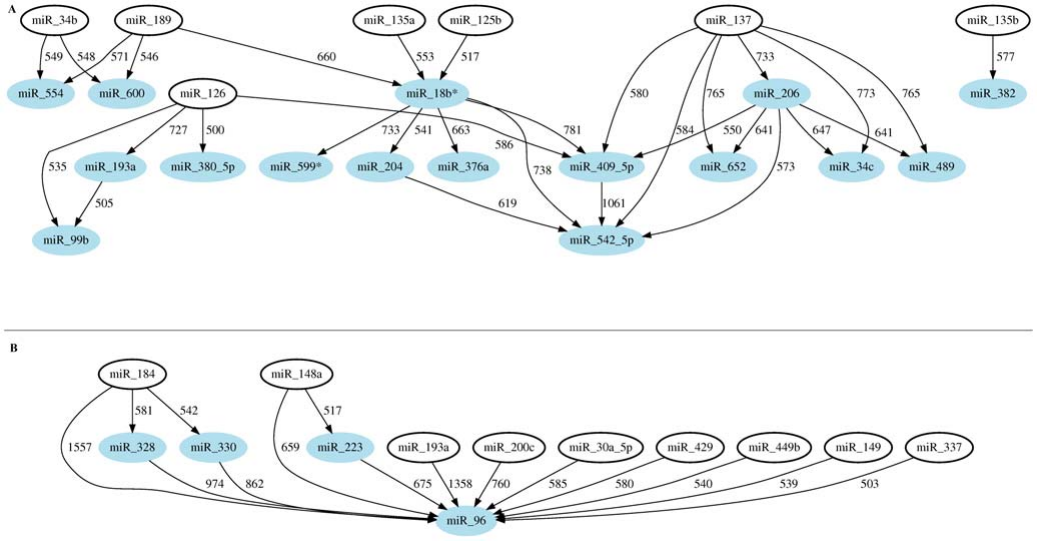
Gene interaction networks from the qPCR data. A: Relapse versus remission status. B: Remission versus control status. Numbers represents robustness score (see material and methods for details). The genes that had at least one parent had been noted with a shaded blue oval.

A coexpression network analysis was performed to obtain information about the relationships between the different miRNA (as explained in material and methods). Two interaction networks were built according to the studied groups;

- **relapse versus remitting**, in order to obtain information about the relapse phenomena in the patients
- **remission versus controls**, in order to obtain information about the Remission stage. (Fig. 3A-B)

From these analyses of the expression data (t-test, SU and coexpression networks), we selected 7 miRNA in which we performed further analysis (see Table 2);

- We chose three miRNA, ***hsa-miR-18b***, ***hsa-miR-493 and hsa-miR-599***, because they reach the significance level in the corrected t-test used to compare relapse status with control samples.
- We selected four miRNA, ***hsa-miR-148a***, ***hsa-miR-184***, ***hsa-miR-193a and hsa-miR-96***, coming from the network analysis that differentiates between remission and control groups. From this network we chose the miRNA with the higher degree (hsa-miR-96), the two with arcs showing the highest robustness values (hsa-miR184 with 1557 robustness scores and hsa-miR-193a with 1358 robustness scores) and the other parent of hsa-miR96, the hsa-miR-148a.

**Table 2:** Selected microRNA based in qPCR experimental data.

Relapse	Remitting	Selected by	Gene ID	chromosome
hsa-mir-18b		**FDR corrected T-test**	547033	Xq26.2
hsa-miR-493			574450	14q32.31
hsa-mir-599			693184	8q22.2
	hsa-miR-96	**co-expression networks**	407053	7q32.2
	hsa-miR-184		406960	15q25.1
	hsa-miR-148a		406940	7p15.2
	hsa-miR-193a		406968	17q11.2

### Validation of the miRNA expression

To validate these results we studied the following three miRNA: miR-18b, miR-96 and miR-599, in an independent set of samples (group C). We choose two miRNA coming from the T-test analysis, and another one coming from the coexpression network.

miR-18b and miR-599 were up-regulated four and five times more in the relapse group than in the controls. For miR-96 we obtained no differences in the expression between the groups.

### Sequencing of the miRNA genes

To discard a DNA conformation effect in the expression of the miRNA, we sequence three genes (hsa-miR-18b, hsa-miR-493 and hsa-miR-96) in the 21 studied samples and in an additional group of 40 samples. We choose randomly two genes characteristic of the relapse group (hsa-miR-18b and hsa-miR-493) and another from the remitting group (hsa-miR-96). No polymorphisms had been found in the sequenced samples.

### miRNA targets

In order to provide a biological interpretation of our findings, we searched the predicted targets of each relevant miRNA in three different databases; **mirbase targets v5**, **Targetsan v4.2** [28]–[30] and **Pictar** [31]. Table 3 lists the number of predicted targets for these miRNA according to each database (two searches with different confidence thresholds were performed in **mirbase**). A complete list of the targets could be found in table S5)

**Table 3:** Predicted number of genes targets for each miRNA in three databases.

	mirbase		Pictar	Targetscan	Common	
	p<0.05	p<0.005				
***18***	*775*	*327*	*151*	*149*	*14*	
***599***	*783*	*185*	*X*	*173*	*11*	Relapse vs Control
***493***	*866*	*244*	*X*	*496*	*3*	
**96**	909	361	698	592	57	
**184**	819	289	22	17	3	Non relapse vs Control
**193**	918	353	429	434	46	
	819	362	134	208	14	

The column labeled as “common” represents the common predicted targets in the three databases. The Gene symbol of these target genes could be found in supplementary methods, table 3.

Theoretically, these miRNA should inhibit the expression of a certain number of target genes. The databases offer predicted information about the targets, but there are few experimental results to support it. In our analysis we took a conservative approach, taking as target genes only the common results from the three different prediction algorithms (in the ***mirbase*** case we selected the p<0.005 column).

To validate these results, we tested the expression of four selected targets of the miRNA more representative of each group in blood from an independent sample set (GroupB), but we saw no statistical differences in these expression pattern. Table S6 presents the miRNA that target these genes and the group in which it is expected to be down-regulated.

Since miRNA are highly conserved across species [32], [33], we used the murine EAE model to validate our findings. To this end, we mined a large multi-tissue, longitudinal gene expression profiling dataset in mouse EAE Lymph Node [9] and Spinal cord [34], focusing on targets of those miRNA differentially expressed in our cohort of MS patients. Briefly, EAE was induced in 84 female NOD mice by s.c. injection into their lower flanks with MOG35–55 peptide emulsified in CFA containing 4 mg/ml *Mycobacterium tuberculosis* (Difco). Immediately thereafter and 48 h later, an i.v. injection of 350 ng of *Bordetella pertussis* toxin was administered to the animals. The control group consisted of another 26 female mice treated with the same protocol except MOG peptide. Samples were extracted at different time points obtaining a longitudinal model of the disease. In each extraction RNA from Spinal cord and lymph node were obtained. The animals were clinically scored every days during the experiment and were classified in four groups: without clinical symptoms, Starting with the symptoms, in the peak of the disease and recovering from the peak.

In order to check whether our target selected genes were really related with the disease, we created a group of 11 randomly selected miRNA from those that were not differentially expressed in our first analysis.

We compared the expression of the target genes (see table 3) for the two groups (7 miRNA from our data and 11 miRNA randomly choose) at the peak of the disease and at the recovering stages between the control and the EAE group.

The target genes were classified the target genes in four groups: not found in the dataset, up-regulated, down-regulated and equally-expressed.

**Figure 4:**
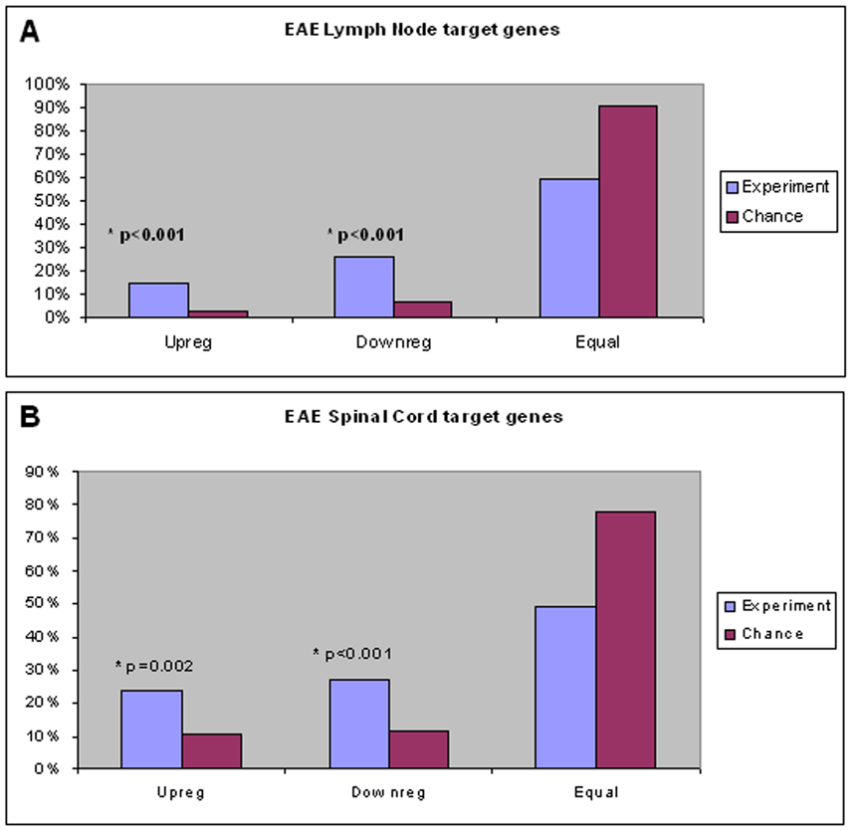
Percentage of the targets founded in the EAE experiment. The founded targets were grouped in up-regulated, down-regulated and equally regulated between the EAE group and the control. The data from the 7 selected miRNA are presented in blue and the data from the randomly selected 11 miRNA in pink

The results are summarized as percentages in Fig. 4. A chi-square analysis was performed between the groups. The figure shows the results of the analysis for the selected genes (in blue) and for the randomly selected group (in pink). The analyzed target-genes were differentially distributed (p<0.001) between experimental and random group in the up-regulated and down-regulated class.

A pathway analysis was conducted in Panther [35] database for all miRNA (experimental and random). A resume of the methods used for panther are available in Supplementary data as Text S1. Out of the seven miRNA targets (miR-18b, miR-599, miR-493, miR-184, miR-148a, mir-96 and miR-193a) only targets of miR-96 appeared significantly enriched in 8 pathways (see table 4 for miR96 results. All the data could be found at table S7).

**Table 4:** Pathway analysis of the hsa-miR-96 targets.

Pathway_hsa-miR-96 targets	NCBI	96	expected	ratio	P-value
**Muscarinic acetylcholine receptor 1 and 3 signaling pathway**	62	5	0.14	35.7	5.39E-05
**Alpha adrenergic receptor signaling pathway**	29	3	0.06	50.0	6.88E-03
**Unclassified**	22436	39	50.29	0.8	1.01E-02
**Endothelin signaling pathway**	98	4	0.22	18.2	1.23E-02
**Interleukin signaling pathway**	194	5	0.43	11.6	1.29E-02
**Wnt signaling pathway**	348	6	0.78	7.7	2.18E-02
**Histamine H1 receptor mediated signaling pathway**	43	3	0.1	30.0	2.19E-02
**Metabotropic glutamate receptor group I pathway**	44	3	0.1	30.0	2.35E-02
**Angiotensin II-stimulated signaling through G proteins and beta-arrestin**	53	3	0.12	25.0	4.04E-02

## Discussion

We identified three miRNA (hsa-mir-18b, hsa-mir-493 and hsa-mir-599) showing differential expression between MS patients experiencing a relapse and controls. Classic parametric tests did not detect differentially expressed miRNA between samples from patients in remission vs controls, from MS vs controls or from relapse vs remission. However, a network-based approach identified 4 miRNA (hsa-mir-96, hsa-mir-148a, hsa-mir-184 and hsa-mir-193) that could be interesting candidates related with the remission stage.

According to the miRNA function, we hypothesized that if a given miRNA was over-expressed in a particular group of samples, the predicted targets of this miRNA should be down-regulated. To check this hypothesis, although this is an indirect approach, we analyze the expression data coming from a longitudinal dataset in mouse EAE model. Interestingly, in this model the target genes of all 7 differentially expressed miRNA appeared significantly down-regulated more times in the targets selected by our experiment than in a random target list. However, we observed a similar effect for the up-regulated genes. These genes may be being downregulated in a translational form and the upregulation of the mRNA could be a retroactive mechanism to valance that.

A biological interpretation of miRNA function in MS is complicated by the fact that most of the miRNA targets are predicted from bioinformatics analysis and are not yet validated in biological studies. To enhance our confidence, we only worked with consensus targets from the three public miRNA databases.

### Patients in relapse status

A t-test identified three differentially expressed genes between relapse and control samples. We would expect the same differences between relapse and remitting groups, however mir-18b and mir-599, but not mir-493, were up-regulated during a relapse, showing a trend that did not reach significance after FDR correction. Moreover in the relapse versus remitting network, two of these genes, miR-18b and miR-599 appear to be correlated with a direct probabilistic relationship (with a 733 robustness score). The expression of these two genes have been validated in an independent set of samples. Taken all together, these results suggest that miR-18b and miR-599 are related in some way to the mechanisms of the relapse. Their role remains unclear, but should be related with the regulation of the proposed target genes.

In the analyses of the pathways in which the target genes of these two miRNA are implicated, neither the target genes of each miRNA individually nor the target genes in common between both miRNA give significant results.

The study of the target genes in patients showed no clear inhibition, as it might be expected, perhaps because regulation of the miRNA is occurring at the translational level rather than at the expression level.

These results support the idea that the expression of the miRNA could be useful as a biomarker of the relapse status.

### Patients in remitting status

The proposed network obtained in the comparison between samples from patients in remission and control samples identified four miRNAs likely to be implicated in the relapses (hsa-miR-148a, hsa-miR-184, hsa-miR-193a and hsa-miR-96). The results suggest that hsa-miR-96 could be an important candidate for further studies. hsa-miR-96 is first in the SU ranking when remitting and control groups are compared. Although the classic qPCR analysis of the expression of this gene gave no significant differences, we note that this gene is more highly expressed in remitting samples than in controls, and less in relapse samples than in remitting. The data from the SU analysis, the network relations and the trend in the qPCR data suggests that has-miR-96 might be characteristic of the remitting phase of the disease. In the validation with an independent set the results of the qPCR data are the same: no differences between the groups but a similar trend in the data.

A Gene Ontology analysis with the target-genes of miR-96 gave a list of 8 categories that reached significant level. As could be expected, within this list we found a classic immune-associated pathways such as *Interleukin signaling pathways*. Two other pathways, the *Metabotropic glutamate receptor group I pathway* and the *Muscarinic acetylcholine receptor 1 and 3 signaling pathway*, related with Glutamate, are also present. Glutamate has been widely related with pathological mechanisms of the MS such as exocitotoxicity [36], [37]. Although these pathways have been more extensively described in the CNS, they may well play a role in activated T-cells.

Another significant GO category pointed toward the *Wnt signaling pathway*. Wnt has been proposed as an important player in the development of effector T-cells and in the activation of the regulatory T-cell [38].

These miRNA and these pathways could be good candidates in further studies about biomarkers and to understand the etiology of the MS

### MS and miRNA

A relationship between miRNA expression and MS is not unexpected as some of the functions attributed to the miRNA include stress response, immunomodulation [39], [40] and neuroprotection [17]. Moreover, bioinformatics-based predictions have suggested that 30% of the human genes are regulated by miRNA [41]. We therefore hypothesize that a sizeable proportion of the mRNA differentially expressed between samples from patients during a relapse and during remission could be regulated by miRNAs.

Our results support the role of miRNA expression patterns in MS. The reliability of the data is sustained by the different statistical approaches, by validation in an independent cohort of samples and by the congruent results, both in the gene ontology analysis and in the animal model analysis.

Although these studies should be replicated in a larger cohort of samples, here we describe a list of miRNAs that could be good candidates in future biomarker studies in MS and at least two more with potential to be good markers to characterize the relapse status.

## Supporting Information

Table S1 Clinical description of the patients. Tev: Time of evolution (years). EDSS: Expanded Disability Status Score. Te: Time from the relapse onset and the blood extraction (in days)
(0.03 MB DOC)

Table S2 Taqman probes distribution in the Taqman Low density array (www.appliedbiosystem.com)
(0.05 MB XLS)

Table S3 DCT data from the TLDA analysis. The data comes from the different comparisons: MS (relapse and remitting) vs Controls; Relapse (Relap) vs controls; remitting(Remitt) vs controls and relapse vs remitting
(0.32 MB DOC)

Table S4 Complete data from the non-parametrical statistical analysis
(0.15 MB XLS)

Table S5 Complete list of the miRNA predicted targets
(0.05 MB XLS)

Table S6 Target genes studied with their gene ID, the miRNA that binds to the gene, the group in which these genes are expected to be down-regulated and the Geneglobe Assay code.
(0.03 MB DOC)

Table S7 Data from the pathway analysis conducted by panther with the predicted gene target lists from each miRNA. Two different groups of miRNA were studied; coming from the experiment and coming from the chance group
(0.05 MB DOC)

Text S1 Resume of the panther software methods
(0.03 MB DOC)
