# Supplementary material for: Differential Micro RNA Expression in PBMC from Multiple Sclerosis Patients
Source: PLoS One. 2009 Jul 20;4(7):e6309. doi: 10.1371/journal.pone.0006309 (PMC2708922; doi:10.1371/journal.pone.0006309)
Supplement: Table S1 — Clinical description of the patients. Tev: Time of evolution (years). EDSS: Expanded Disability Status Score. Te: Time from the relapse onset and the blood extraction (in days) (0.03 MB DOC) [file pone.0006309.s001.doc]

**Supp T-1.** Clinical description of the patients. Tev: Time of evolution (years). EDSS: Expanded Disability Status Score. Te: Time from the relapse onset and the blood extraction (in days)
