# Supplementary material for: Differential Micro RNA Expression in PBMC from Multiple Sclerosis Patients
Source: PLoS One. 2009 Jul 20;4(7):e6309. doi: 10.1371/journal.pone.0006309 (PMC2708922; doi:10.1371/journal.pone.0006309)
Supplement: Table S6 — Target genes studied with their gene ID, the miRNA that binds to the gene, the group in which these genes are expected to be down-regulated and the Geneglobe Assay code. (0.03 MB DOC) [file pone.0006309.s006.doc]

|  | ***Gene ID*** | ***miRNA*** | ***Expected to be down regulated in:*** | ***Assay code*** |
| --- | --- | --- | --- | --- |
| ***ARHGEF12*** | 23365 | 96 / 148 / 193 | Remitting group | QT00006762 |
| ***CELSR2*** | 1952 | 96 | Remitting group | QT00010948 |
| ***TAOK3*** | 51347 | 599 | Relapse group | QT00059843 |
| ***GAB1*** | 2549 | 18b | Relapse group | QT00014154 |

Table 6: Target genes studied with their gene ID, the miRNA that binds to the gene, the group in which these genes are expected to be down-regulated and the Geneglobe Assay code.
