# Supplementary material for: Differential Micro RNA Expression in PBMC from Multiple Sclerosis Patients
Source: PLoS One. 2009 Jul 20;4(7):e6309. doi: 10.1371/journal.pone.0006309 (PMC2708922; doi:10.1371/journal.pone.0006309)
Supplement: Text S1 — Resume of the panther software methods (0.03 MB DOC) [file pone.0006309.s008.doc]

Resume of the panther software, more information could be found in the web site (www.pantherdb.org)

- The first column contains the name of the PANTHER classification category.
- The second column contains the number of genes in the reference list (from NCBI) that map to this particular PANTHER classification category.
- The third column contains the number of genes in your uploaded list that map to this PANTHER classification category.
- The fourth column contains the [expected value](http://www.pantherdb.org/tips/tips_binomial.jsp#Expected_Value), which is the number of genes you would expect in your list for this PANTHER category, based on the reference list.
- The fifth column represents a ratio between the expected and the observed. Greater than 1 indicates over-representation of this category in your experiment: you observed more genes than expected based on the reference list (for this category, the number of genes in your list is greater than the expected value). Conversely, lower than 1 indicates under-representation.
- The sixth column is the [p-value as determined by the binomial statistic](http://www.pantherdb.org/tips/tips_binomial.jsp#P-Value_calculated). This is the probability that the number of genes you observed in this category occurred by chance (randomly), as determined by your reference list. A small p-value indicates that the number you observed is significant and potentially interesting. A cutoff of 0.05 is recommended as a starting point.

### Expected Value

The expected value is the number of genes you would expect in your list for a particular PANTHER category, based on the reference list. If for this biological process you observe more genes in your uploaded list than expected, you have an over-representation of genes involved in chromatin packaging and remodeling. If you observe fewer genes than expected, you have an under-representation.

### P-Value calculated by the Binomial statistic

The binomial statistic is a commonly used statistic. In the binomial test we assume that under the NULL hypothesis, genes in the uploaded list are sampled from the same general population as genes from the reference set, i.e. the probability p(C) of observing a gene from a particular category C in the uploaded list is the same as in the reference list. We first estimate the probability p(C) from the reference set assuming that it is large and representative:

p(C)=n(C)/N,

where n(C) is the number of genes mapped to category C, and N is the total number of genes in the reference set. We then use the above estimate to find the p-value: the probability of observing k(C) genes (or a more extreme number) in the uploaded list of size K. Under the NULL hypothesis, the number of genes of mapped to C is distributed binomially with probability parameter p(C) and thus the p-value would be


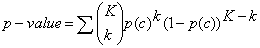


 where the sum runs from k(C) to K in the case of over-representation (i.e. when the number of observed genes k(C) is greater than expected p(C)*K under the NULL hypothesis), and 0 to k(C), in the case of under-representation (i.e. when k(C) is smaller than p(C)*K).

When developing this analysis tool, we tested using both the Chi-Square and Binomial statistical tests. We decided to use the Binomial, since the Chi-Square is not as accurate when the population sizes or the expect number is small.
